# Pneumonia severity scores in resource poor settings

**DOI:** 10.15172/pneu.2014.5/481

**Published:** 2014-12-01

**Authors:** Jamie Rylance, Peter Waitt

**Affiliations:** 15 Department of Respiratory Medicine, Clinical Sciences Centre, University Hospital Aintree, Lower Lane, Liverpool, L9 7AL UK; 25 Department of Acute Medicine, Arrowe Park Hospital, Arrowe Park Road, Wirral, Merseyside, CH49 5PE UK

**Keywords:** pneumonia, severity of illness index, developing countries, health resources, operatons research

## Abstract

Clinical prognostc scores are increasingly used to streamline care in well-resourced setngs. The potental benefts of identfying patents at risk of clinical deterioraton and poor outcome, delivering appropriate higher level clinical care, and increasing efciency are clear. In this focused review, we examine the use and applicability of severity scores applied to patents with community acquired pneumonia in resource poor setngs. We challenge clinical researchers working in such systems to consider the generalisability of existng severity scores in their populatons, and where performance of scores is suboptmal, to promote eforts to develop and validate new tools for the beneft of patents and healthcare systems.

## 1. Introduction

Severity scores are designed to identify patients at high risk of adverse outcome. They allow resources to be concentrated on such patients, with a strong emphasis on early intervention. Disease specific scores, as described here for community acquired pneumonia (CAP), may additionally direct clinical decisions regarding treatment and discharge.

Severity scores include factors strongly associated with adverse outcome. Combining multiple factors improve the identification of patients at highest or lowest prognostic risk. Given a well-defined cohort, statistical methods make the generation of such scores straightforward. In order to be clinically useful, they should be widely applicable, objectively measurable, and simple. Crucially, the implementation of severity score-associated pathways and treatment plans must have a proven positive impact for patients. This challenge requires an understanding of the benefits and limitations of severity scores by clinicians and service planners. This review highlights the current available tools, particularly those of relevance to resource-limited settings in low and middle income countries (LMICs). We discuss how future efforts might refine and implement such severity scores effectively, and highlight potential limitations of their use, in particular the risk of extrapolation to other diseases without specific validation.

## 2. The purpose of severity scores

Severity scores have been used in clinical practice for decades, but their inclusion into healthcare delivery systems is more recent. Current practice tends to artificially distinguish between early warning scores (EWSs) and severity scores as their current application varies. Both, however, may have four broad aims:

1. To enable junior clinical staff to identify critically unwell patients, and prompt a senior response [1]. For example, this might empower a ward nurse to contact on call doctor out-of-hours, and convey that urgent action may be required
2. To track the severity of a patient’s illness over time, and trigger intervention early in “treatment failure” (track and trigger). For example, relatively unskilled workers can be used to measure and record observations, and identify patients in need of attention with very little medical knowledge
3. To guide initial clinical management e.g. identify patients who could be managed in the community, who require intensive treatment unit care, or to determine whether oral or intravenous antibiotics are most appropriate
4. To enable comparison of quality of care between dissimilar patient populations For example, for auditing the performance of different hospitals.

Generic EWSs tend to focus on the first two goals. Disease specific severity scores, such as those for CAP, are typically promoted as guides to clinical management. These differences are mostly conceptual or historical, and such distinctions might hinder the development or application of future services.

## 3. The perfect severity score

The ideal CAP severity score, universally applied, would simply identify the risk of deterioration in patients, and indicate a proportionate intervention to maximise individual patient outcome and promote efficient service delivery.

Table 1 gives a summary of the perfect performance of a severity score. In practical use, we suggest that severity scores fall short of perfect in three areas: discrimination, application and intervention. The **discrimination** power of a score describes how well the score is calibrated to efficiently separate high and low risk patient groups. Measures of discrimination are given by sensitivity, specificity, positive and negative predictive values. These are discussed separately later. Most published studies focus on the discriminating power of severity scores in either the original cohort (derivation cohort) or new populations (validation cohorts).

**Table 1:** Aiming for perfection — characteristics of an ideal severity score, and practical limitations

Characteristic	Key features	Practical constraints
Simple	Includes routinely recorded data	Limitations of demographic and physiological data
	Easy to calculate	All systems require training at roll-out and later reinforcement.
	Memorable or computer-based tool	Paper and computer systems are limited by availability
Observer independent	Consistency and reliability	Training is required for reliable physiological measurements
		Functioning medical equipment is needed for some variables
Systematic	Comprehensively applied	Scores may be validated for unrealistically well-defined circumstances
	Useful in varied populations	Dissimilar environments and populations require revalidation of existing scores to ensure utility
Specifically applied	Appropriately used in a validated population e.g. suspected pneumonia (CURB-65), gastrointestinal bleed (Blatchford Bleeding Score)	Disease specific scores are quickly unreliable where diagnoses are uncertain, unconfirmed, or over-generalised
Indicates a scale of response	Scores quantitatively reflect outcomes, or urgency. Linearity is ideal i.e. doubling the score indicates the patient is twice as ill	Most trigger scores are calibrated to “all or nothing” outcomes Triage systems are more finely graded and responsive but more complex
For triggering scores:		
Trigger early	Early intervention is a key factor in improving outcome	Timely action in hospital systems requires significant human resources Identifying patients too late to alter outcome is not clinically relevant
Trigger threshold in “Goldilocks” zone	Insensitive trigger misses the opportunities to act Triggering too easily increases workload	High discrimination power is often practically unachievable “Alarm fatigue” leads to reduced staff compliance with procedures

The **application** of severity scores is less straightforward to measure, but describes how well they are incorporated into existing clinical setting. Local implementation should promote consistent and widespread use within an organisation, and should provide resources and support to allow this. Without these, scoring systems remain research tools.

The **intervention** step links the severity score to a meaningful clinical action. Most commonly, this is a trigger to summon senior individuals or liaison with critical care facilities. Pneumonia scores are also commonly used to determine antibiotic choice. For low risk individuals, a useful action might be prompting patient discharge.

Delivering improved outcomes requires attention to all of these three areas. Application and intervention strategies often require systems change: national and local guidelines have begun to address these areas. For example, in the United Kingdom (UK), the British Thoracic Society has established CAP guidelines and, importantly, audit standards by which to judge their implementation [2].

## 4. Severity scores for community acquired pneumonia (CAP) are used to stratify risk in order to guide clinical management

Many severity scoring systems related to CAP have been described, and are summarised in Table 2. For a comprehensive account, a recent systematic review provides full details [11]. These tools range from the easily memorable to extremely complicated, each with a different focus. In common, however, they are all examples of single point “trigger” systems. These contrast with more generic EWSs [12] which operate by “track and trigger”, that is, repeatedly measure the same score to determine both the baseline risk, and early signs of deterioration after admission.

**Table 2:** Severity scores currently used or proposed for community acquired pneumonia

Scoring system [Ref]	Demographic	Comorbidities	Exam		Physiology					Laboratory / Radiology						Details
			Mental state	Other	hr	rr	bp	T	SPO_2_	Urea	FBC	ABG	Alb	Other	CXR	
CURB-65 [3]	Age		✔			✔	✔			✔						1 point each for: confusion; urea >7 mmol; rr ≥30; sbp <90 or dbp ≤60; age ≥65 years.
CRB-65 [3]	Age		✔			✔	✔									As CURB-65, without urea criterion
PSI/PORT [4]	Age^a^	^a^	✔		✔	✔	✔	✔		✔	✔	✔	✔	✔	✔	Complex weighted sum (20 variables, stratifying outcome to 5 risk categories)
SWAT-Bp [5]	Sex			✔			✔	✔								1 point each for: male; wasting; non-ambulatory; T <35 or >38; sbp <90 or dbp <60
Sepsis score^b^ [6]					✔	✔	✔	✔			✔			✔		4 categories: no SIRS^c^; SIRS^c^; severe sepsis^d^; septic shock^e^
ATS 2001 [7]					✔	✔	✔	✔				✔			✔	2 categories: ICU or not
ATS-IDSA [8]			✔		✔	✔	✔	✔		✔	✔	✔			✔	2 categories: ICU or not
SMART-COP [9]	Age		✔		✔	✔	✔		✔			✔	✔		✔	Weighted sum stratifying to 2 categories: ICU or not
SCAP [10]	Age		✔			✔	✔			✔		✔			✔	Weighted sum stratifying to 2 categories: ICU or not

Note: Predecessor systems are omitted for clarity

bp, blood pressure (mmHg); sbp/dbp, systolic/diastolic blood pressure (mmHg); rr, respiratory rate (min^−^^1^); hr, heart rate (min^−^^1^); T, temperature (°C); SpO_2_, blood oxygen saturation (%); FBC, full blood count; ABG, arterial blood gas; alb, albumin; CXR, chest radiograph; SIRS, Systemic Inflammatory Response Syndrome; ICU, intensive care unit.

^a^multiple variables used

^b^not designed as a pneumonia specific score

^c^2 or more of: T <36 °C or >38 °C; hr >90 min^−1^; rr >20 min^−1^; white cell count <4 or >12 10^9^L^−1^

^d^sepsis + evidence of organ dysfunction or hypotension

^e^severe sepsis resistant to fluid resuscitation

The PSI/PORT (Pneumonia Severity Index/Patient Outcomes Research Team) was published in 1997 [4], and can identify low risk patients by calculation of a weighted score based on 20 variables. It remains the research standard [13], but requires a broad range of laboratory tests to implement. CURB-65 (Confusion, Urea, Respiratory rate, Blood pressure, Age > 65 years) and CRB-65 (Confusion, Respiratory rate, Blood pressure, Age > 65 years) severity scores for CAP are designed to more simply stratify patients according to risk, including those at low and high extremes (prompting consideration of out-patient and intensive care unit level care respectively). CURB-65 and CRB-65 have been widely validated in high income countries and predict 30 day mortality. However, of 40 studies included in a systematic review of articles published between 1980 and 2009 [14], only one study was derived from a LMIC. Given the paucity of evidence, recent validation efforts in new settings are welcome [15]. The SWAT-Bp (male Sex, Wasting, non-Ambulatory, Temperature, Blood pressure) score was derived from an inpatient population in Malawi where CRB-65 performs less well than in Europe [5]. Preliminary data suggests internal validity [16].

Criteria proposed in the ATS 2001 (American Thoracic Society) pneumonia guidelines [7], ATS-IDSA (American Thoracic Society-Infectious Disease Society of America) [8], SMART-COP (Systolic blood pressure, Multilobar infiltrate, Albumin, Respiratory rate, Tachycardia, Confusion, low Oxygen, low PH) [9] and SCAP (Severe Community-Acquired Pneumonia) [10] are derived from, and used in, high-income environments where ventilatory support and vasopressor use are common. These criteria aim to identify patients who should be considered for intensive care unit admission. Their successful adoption will mean that new severity scores in this setting should be validated against objective outcomes rather than “need for critical care” in order to prevent circularity.

Sepsis scores, although not deliberately calibrated for use in CAP, have similar, if slightly reduced, discriminatory value [17]. This suggests that all of these tools are more generally measuring a pathological systemic inflammatory response [18].

## 5. Validation of severity scores is necessary in “new” populations

Even within one health system, clinicians should be aware of the scope and applicability of severity scores. Some systems continue to work well outside of their original disease definitions (CURB-65 predicts severity in chronic obstructive pulmonary disease exacerbation in the UK [19]). Conversely, even when appropriately used, CURB-65 has some limitations. For example, disease severity is underestimated in relatively young (<50 years) and old (>85 years) patients [20,21]. This is a problem where the ‘scoring’ variables (here, age) diverge significantly from the demographic represented in the derivation cohort. Where patterns of disease are atypical, more generalised scores may be more accurate, if unwieldy. One example is the superiority of the APACHE II (Acute Physiology and Chronic Health Evaluation II) score over CURB-65 in methicillin-resistant *Staphylococcus aureus* pneumonia [22].

Where there are more significant differences in environment, disease prevalence or patient characteristics, repeated validation becomes even more important. This is illustrated by comparing performance of the CRB-65 score in patients from Germany [23] and Malawi [5] (Table 3). In sub-Saharan Africa, CAP incidence is higher, median age is lower, human immunodeficiency virus infection is more common, and diagnostics more limited. The discrimination power of the score (sensitivity and specificity) is altered. Negative predictive value (NPV) and positive predictive value (PPV) are particularly sensitive to the relative frequency of disease, and are also the most important descriptors of the real world usefulness of the system. For example, to identify “low risk” patients, a high NPV is critical. Using a threshold value of >2 in the German cohort has an NPV of 97%, that is, only 3% of individuals are misclassified as low risk. If adopted in Malawi, the corresponding NPV is 85%, meaning that the same system will be falsely reassuring in 15% of cases. Similar problems are faced with EWSs [24]. Adoption of guidelines from other settings without local revalidation may therefore lead to increased staff workload, inadequate clinical care or misdirection of limited resources. In the example above, many patients could be discharged who were at significant clinical risk of deterioration. In resource limited settings, the likelihood of poor outcome is increased by the high opportunity costs of readmission (e.g. time, transport, geographical inaccessibility, dependence on family for funds).

**Table 3:** An example of the loss of discriminating power in cohorts with different characteristics

	Severity Scores					
	CRB-65 = 0		CRB-65 ≥2		CRB-65 ≥3	
	Germany	Malawi	Germany	Malawi	Germany	Malawi
True Positive	0	0	50	16	13	3
False Positive	0	0	366	38	53	4
True Negative	375	60	1,034	158	1,347	192
False Negative	0	4	27	28	64	41
PPV (%)	N/A	N/A	12	30	19	43
NPV (%)	50	94	97	85	95	82
Sensitivity (%)	0	0	65	36	17	7
Specificity (%)	100	1	74	81	96	98

Note: CRB-65 scores have been applied to patients from Germany [23] and from Malawi [5]. Numbers indicate the number of patients in each category.

NPV, negative predictive value; PPV, positive predictive value; N/A, incalculable

### 5.1 Improving severity score performance in new settings

Strong risk factors are consistently incorporated into severity scores, such as indices of blood pressure, heart rate and conscious level (Table 2). It is unlikely that many novel physiological risk factors will be found, although mid-upper arm circumference does show promise in the Malawi study. Generic markers of infirmity such as inability to walk have been useful, and under-reported [24]. Refinement of existing scores, rather than reinvention, may therefore be most appropriate. Historical factors might be helpful in this way and should be investigated, for example, prior use of antibiotics. Other patient information offers the opportunity to tune severity scores to local disease prevalence. In one study in Kenya with endemic rates of tuberculosis, 9% of acute respiratory disease consistent with pneumonia was found to be mycobacterial [25]. In these circumstances, haemoptysis and chronicity might be investigated, or possibly incorporated into clinical pathways. Lastly, hypoxia as measured by peripheral oxygen saturations (SpO_2_) is becoming widely available. In well-resourced settings, its use can improve on CURB-65 [26]. Even where oxygen availability may be severely limited, the use of SpO_2_ as a marker of severity rather than a criterion for supplemental oxygen may be worthwhile, but the data are lacking.

As such, we cannot currently recommend any of the available pneumonia severity scores in resource-limited settings such as Malawi. However, there are huge potential gains where improvements can be made, and relevant research is urgently needed.

## 6. Judicious implementation of clinical systems based on severity scores has significant advantages

The introduction of severity scores may directly improve clinical care. This could be a direct effect of identifying critically unwell patients. By the recording of pertinent severity markers, physicians are explicitly encouraged to assess the severity of patient illness. Incorporation of severity scores into undergraduate and postgraduate teaching gives physicians-in-training a practical framework on which to base their clinical decisions, especially when junior medical staff are frequently professionally isolated in rural areas. There are also potential benefits from standardising and auditing practice, making it easier to identify meaningful trends in patient outcome over time, or between facilities.

Indirectly, the incorporation of severity scores into quality improvement schemes can focus efforts on staff education, or hospital structures. For example, even where ward level nurse supervision is difficult, it is possible to cohort the most unwell patients in proximate areas, thereby improving the likelihood of timely medical input.

Implementation of “antimicrobial stewardship” tools are likely to have wider impact [27], and may conceivably have at their heart severity scores for common diseases such as pneumonia. For example, prescription of broad spectrum intravenous antimicrobials might be limited to patients with high severity scores.

Potential hazards lie in increasing administrative overhead, and reducing the flexibility of the healthcare system. To mitigate against these potential disadvantages, clinicians should understand the scope of the severity scores they are using, and the appropriateness of the score to their patient group. More pragmatically, scores which are simple, memorable, and require limited laboratory data (such as CURB-65) are likely to be the most successful.

### 6.1 Maximising impact — learning lessons from early warning score (EWS) implementation

EWSs were initially conceived to improve identification of deteriorating patients, and to facilitate nurses in triggering early senior medical reviews. In the UK, they have been widely adopted, although the use of multiple systems and poor early reliability, and sensitivity has been problematic [28]. Clinicians have also expressed concerns over fragmentation of clinical work, and these shortcomings have been recognised. This has prompted action to standardise systems across different hospitals, and to promote “task shifting” — transferring defined tasks from doctors to other healthcare professionals — to optimise human resource allocation.

Where CAP severity scores identify large numbers of patients at high risk, there may be a similar effect to EWS systems. The demand for resources is likely to increase, and in resource limited settings this may frequently highlight shortfalls in oxygen availability [29], or critical care provision [30,31]. It is important that implementing CAP interventions at the expense of other essential services does not have an overall negative impact. However, prioritization of the critical unwell patient with CAP is likely to be key to improving outcomes. Severity scores, resource allocation (particularly human resources) and interventions should therefore be locally appropriate. Future research studies assessing their impact should examine the healthcare delivery in a broad context, including both patient outcome data and resource implicaions.

## 7. Conclusions and future directions

Severity risk scores can be an excellent tool to enable identification of both patients at risk of deterioration, and patients at lower risk who may not require hospital admission at all. For optimal use, their limitations must be understood, as must the population within which they were derived. To aid clinicians in resource-poor settings, two types of severity score will ideally develop.

Firstly, risk stratification tools should be validated, by refinement of existing systems (e.g. CURB-65) to improve their performance in new populations. This will be the most cost-effective option to implement. Secondly, the development of ‘track and trigger’ systems would additionally allow the identification of deteriorating patients, but carries resource implications in the repeated measurement of physiological markers. Further operational research is required following implementation of any risk score system to demonstrate its overall benefits. In the same way as CURB-65 performs well in many high income countries, it is possible that alternative systems might be suitable for a broad range of LMICs. This would allow standardised interventions, including “bundles of care”, analogous to the adult triage system proposed by the World Health Organization as part of the Integrated Management of Adolescent and Adult Illness project [32]. Where sepsis and CAP scores work similarly, it was proposed that a more generic application of risk stratification could be incorporated into rapid treatment protocols, and implemented by healthcare workers other than doctors. Using broadly applicable severity markers in this setting could help the development of wider triage systems, which currently do not exist in many low resource setngs.

### Key Points:

Scoring systems are used to focus resources.

They should be validated in a population in which they are to be used.

“Trigger” scores should prompt action which is likely to improve prognosis.

Trade-offs in sensitivity and specificity are unavoidable: with inappropriate implementation, severity scores can increase workload without improving outcomes.

## References

[1] Morgan RJ, Wright MM (2007). In defence of early warning scores. Br J Anaesth.

[2] Lim WS, Baudouin SV, George RC, Hill AT, Jamieson C, Le Jeune I (2009). Pneumonia Guidelines Commitee of the BTS Standards of Care Commitee. BTS guidelines for the management of community acquired pneumonia in adults: update 2009. Thorax.

[3] Lim WS, van der Eerden MM, Laing R, Boersma WG, Karalus N, Town GI (2003). Defning community acquired pneumonia severity on presentaton to hospital: an internatonal derivaton and validaton study. Thorax.

[4] Fine MJ, Auble TE, Yealy DM, Hanusa BH, Weissfeld LA, Singer DE (1997). A predicton rule to identfy low-risk patents with community-acquired pneumonia. N Engl J Med.

[5] (2013). PLoS ONE.

[6] American College of Chest Physicians/ (1992). Society of Critcal Care Medicine Consensus Conference: defnitons for sepsis and organ failure and guidelines for the use of innovatve therapies in sepsis. Crit Care Med.

[7] Ewig S, Ruiz M, Mensa J, Marcos MA, Martnez JA, Arancibia F (1998). Severe community-acquired pneumonia. Assessment of severity criteria. Am J Respir Crit Care Med.

[8] Mandell LA, Wunderink RG, Anzueto A, Bartlet JG, Campbell GD, Dean NC (2007). Infectous Diseases Society of America; American Thoracic Society. Infectous Diseases Society of America/American Thoracic Society consensus guidelines on the management of community-acquired pneumonia in adults. Clin Infect Dis.

[9] Charles PG, Wolfe R, Whitby M, Fine MJ, Fuller AJ, Strling R (2008). Australian Community-Acquired Pneumonia Study Collaboraton. SMART-COP: a tool for predictng the need for intensive respiratory or vasopressor support in community-acquired pneumonia. Clin Infect Dis.

[10] España PP, Capelastegui A, Quintana JM, Bilbao A, Diez R, Pascual S (2010). Validaton and comparison of SCAP as a predictve score for identfying low-risk patents in community-acquired pneumonia. J Infect.

[11] Mart C, Garin N, Grosgurin O, Poncet A, Combescure C, Carballo S (2012). Predicton of severe community-acquired pneumonia: a systematc review and meta-analysis. Crit Care.

[12] Royal College of Physicians. (2012). Natonal Early Warning Score (NEWS): Standardising the assessment of acute-illness severity in the NHS.

[13] Aujesky D, Fine MJ (2008). The pneumonia severity index: a decade afer the inital derivaton and validaton. Clin Infect Dis.

[14] Chalmers JD, Singanayagam A, Akram AR, Mandal P, Short PM, Choudhury G (2010). Severity assessment tools for predictng mortality in hospitalised patents with community-acquired pneumonia. Systematc review and meta-analysis. Thorax.

[15] Alavi-Moghaddam M, Bakhshi H, Rezaei B, Khashayar P (2013). Pneumonia severity index compared to CURB-65 in predictng the outcome of community acquired pneumonia among patents referred to an Iranian emergency department: a prospectve survey. Braz J Infect Dis.

[16] Buss I, Birkhamshaw E, Magadoro I, Innes M, Rylance J, Wait P (2012). Validaton of a new index to predict mortality from community-acquired pneumonia in Malawi: the SWAT-BP score 43rd World Conference. IUATLD.

[17] Barlow G, Nathwani D, Davey P (2007). The CURB65 pneumonia severity score outperforms generic sepsis and early warning scores in predictng mortality in community-acquired pneumonia. Thorax.

[18] Howell MD, Donnino MW, Talmor D, Clardy P, Ngo L, Shapiro NI (2007). Performance of severity of illness scoring systems in emergency department patents with infecton. Acad Emerg Med.

[19] Chang CL, Sullivan GD, Karalus NC, Mills GD, McLachlan JD, Hancox RJ (2011). Predictng early mortality in acute exacerbaton of chronic obstructve pulmonary disease using the CURB65 score. Respirology.

[20] Chalmers JD, Singanayagam A, Hill AT (2008). Predictng the need for mechanical ventlaton and/or inotropic support for young adults admited to the hospital with community-acquired pneumonia. Clin Infect Dis.

[21] Parsonage M, Nathwani D, Davey P, Barlow G (2009). Evaluaton of the performance of CURB-65 with increasing age. Clin Microbiol Infect.

[22] Kollef KE, Reichley RM, Micek ST, Kollef MH (2008). The modifed APACHE II score outperforms Curb65 pneumonia severity score as a predictor of 30-day mortality in patents with methicillin-resistant Staphylococcus aureus pneumonia. Chest.

[23] Bauer TT, Ewig S, Marre R, Sutorp N, Welte T, Group CS (2006). CAPNETZ Study Group. CRB-65 predicts death from community-acquired pneumonia. J Intern Med.

[24] Rylance J, Baker T, Mushi E, Mashaga D (2009). Use of an early warning score and ability to walk predicts mortality in medical patents admited to hospitals in Tanzania. Trans R Soc Trop Med Hyg.

[25] Scot JA, Hall AJ, Muyodi C, Lowe B, Ross M, Chohan B (2000). Aetology, outcome, and risk factors for mortality among adults with acute pneumonia in Kenya. Lancet.

[26] Sanz F, Restrepo MI, Fernández E, Mortensen EM, Aguar MC, Cervera A (2011). Neumonía Adquirida en la Comunidad de la Comunidad Valenciana Study Group. Hypoxemia adds to the CURB-65 pneumonia severity score in hospitalized patents with mild pneumonia. Respir Care.

[27] Hurst JM, Bosso JA (2013). Antmicrobial stewardship in the management of community-acquired pneumonia. Curr Opin Infect Dis.

[28] Gao H, McDonnell A, Harrison DA, Moore T, Adam S, Daly K (2007). Systematic review and evaluation of physiological track and trigger warning systems for identifying at-risk patients on the ward. Intensive Care Med.

[29] Belle J, Cohen H, Shindo N, Lim M, Velazquez-Berumen A, Ndihokubwayo JB (2010). Infuenza preparedness in low-resource setngs: a look at oxygen delivery in 12 African countries. J Infect Dev Ctries.

[30] Riviello ED, Letchford S, Achieng L, Newton MW (2011). Critcal care in resource-poor setngs: lessons learned and future directons. Crit Care Med.

[31] Firth P, Ttendo S (2012). Intensive care in low-income countries—a critcal need. N Engl J Med.

[32] WHO. (2011). IMAI District Clinician Manual: Hospital Care for Adolescents and Adults (Guidelines for the Management of Common Illnesses with Limited Resources — Volume 1).

